# Speech through ears and eyes: interfacing the senses with the supramodal brain

**DOI:** 10.3389/fpsyg.2013.00388

**Published:** 2013-07-12

**Authors:** Virginie van Wassenhove

**Affiliations:** ^1^Cognitive Neuroimaging Unit, Brain Dynamics, INSERM, U992Gif/Yvette, France; ^2^NeuroSpin Center, CEA, DSV/I2BMGif/Yvette, France; ^3^Cognitive Neuroimaging Unit, University Paris-SudGif/Yvette, France

**Keywords:** analysis-by-synthesis, predictive coding, multisensory integration, Bayesian priors

## Abstract

The comprehension of auditory-visual (AV) speech integration has greatly benefited from recent advances in neurosciences and multisensory research. AV speech integration raises numerous questions relevant to the computational rules needed for binding information (within and across sensory modalities), the representational format in which speech information is encoded in the brain (e.g., auditory vs. articulatory), or how AV speech ultimately interfaces with the linguistic system. The following non-exhaustive review provides a set of empirical findings and theoretical questions that have fed the original proposal for predictive coding in AV speech processing. More recently, predictive coding has pervaded many fields of inquiries and positively reinforced the need to refine the notion of internal models in the brain together with their implications for the interpretation of neural activity recorded with various neuroimaging techniques. However, it is argued here that the strength of predictive coding frameworks reside in the specificity of the generative internal models not in their generality; specifically, internal models come with a set of rules applied on particular representational formats themselves depending on the levels and the network structure at which predictive operations occur. As such, predictive coding in AV speech owes to specify the level(s) and the kinds of internal predictions that are necessary to account for the perceptual benefits or illusions observed in the field. Among those specifications, the actual content of a prediction comes first and foremost, followed by the representational granularity of that prediction in time. This review specifically presents a focused discussion on these issues.

## Introduction

**Figure 1:**
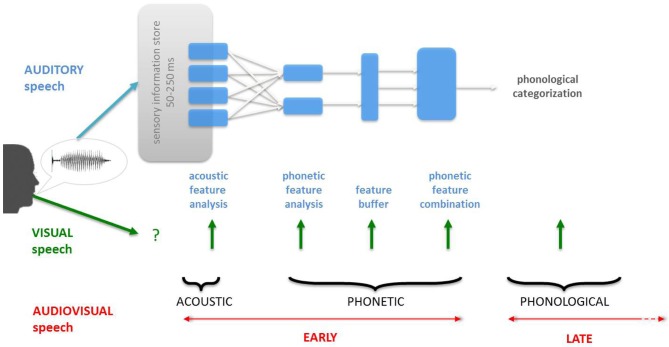
**Classic information-theoretic description of speech processing**. Classic models of speech processing have been construed on the basis of the acoustics of speech, leaving aside the important contribution of visual speech inputs. As a result, the main question in audiovisual (AV) speech processing has been: when does visual speech information integrate with auditory speech? The two main alternatives are before (acoustic or phonetic features, “early” integration) or after (“late” integration) the phonological categorization of the auditory speech inputs (see also Schwartz et al., 1998). However, this model unrealistically frames and biases the question of “when” by imposing a serial, linear and hierarchical processing for speech processing.

In natural conversational settings, watching an interlocutor's face does not solely provide information about the speaker's identity or emotional state: the kinematics of the face articulating speech can robustly influence the processing and comprehension of auditory speech. Although audiovisual (AV) speech perception is ecologically relevant, classic models of speech processing have predominantly accounted for speech processing on the basis of acoustic inputs (e.g., Figure 1). From an evolutionary standpoint, proximal communication naturally engages multisensory interactions i.e., vision, audition, and touch but it is not until recently that multisensory integration in the communication system of primates has started to be investigated neurophysiologically (Ghazanfar and Logothetis, 2003; Barraclough et al., 2005; Ghazanfar et al., 2005, 2008; Kayser et al., 2007, 2010; Kayser and Logothetis, 2009; Arnal and Giraud, 2012). Advances in multisensory research has raised core issues: how early do multisensory integration occur during perceptual processing (Talsma et al., 2010)? In which representational format do sensory modalities interface for supramodal (Pascual-Leone and Hamilton, 2001; Voss and Zatorre, 2012) and speech analysis (Summerfield, 1987; Altieri et al., 2011)? Which neuroanatomical pathways are implicated (Calvert and Thesen, 2004; Ghazanfar and Schroeder, 2006; Driver and Noesselt, 2008; Murray and Spierer, 2011)? In Humans, visual speech plays an important role in social interactions (de Gelder et al., 1999) but also, and crucially, interfaces with the language system at various depth of linguistic processing (e.g., McGurk and MacDonald, 1976; Auer, 2002; Brancazio, 2004; Campbell, 2008). AV speech thus provides an appropriate model to address the emergence of supramodal or abstract representations in the Human mind and to build upon a rich theoretical and empirical framework elaborated in linguistic research in general (Chomsky, 2000) and in speech research, in particular (Chomsky and Halle, 1968; Liberman and Mattingly, 1985).

## Weighting sensory evidence against internal non-invariance

Speech theories have seldom incorporated visual information as raw material for speech processing (Green, 1996; Schwartz et al., 1998) although normal hearing and hearing-impaired populations greatly benefit from looking at the interlocutor's face (Sumby and Pollack, 1954; Erber, 1978; MacLeod and Summerfield, 1987; Grant and Seitz, 1998, 2000). If any benefit for speech encoding is to be gained in the integration of AV information, the informational content provided by each sensory modality is likely to be partially, but not solely, redundant i.e., complementary. For instance, the efficiency in AV speech integration is known to depend not only on the amount of information extracted in each sensory modality but also in its variability (Grant et al., 1998). Understanding the limitations and processing constraints of each sensory modality is thus important to understand how non-invariance in speech signals leads to invariant representations in the brain. In that regards, should speech processing be considered “special?” The historical debate is outside the scope of this review but it is here considered that positing an internal model dedicated to the processing of speech analysis is legitimate to account for (i) the need for invariant representations in the brain, (ii) the parsimonious sharing of generative rules for perception/production and (iii) the ultimate interfacing of the (AV) communication system with the Human linguistic system. As such, this review focuses on the specificities of AV speech not on the general guiding principles of multisensory (AV) integration.

### Temporal parsing and non-invariance

A canonical puzzle in (auditory, visual and AV) speech processing is how the brain correctly parses a continuous flow of sensory information. Like auditory speech, the visible kinematics of articulatory gestures hardly provides non-invariant structuring of information over time (Kent, 1983; Tuller and Kelso, 1984; Saltzman and Munhall, 1989; Schwartz et al., 2012) yet temporal information in speech is critical (Rosen, 1992; Greenberg, 1998). Auditory speech is typically sufficient to provide a high level of intelligibility (e.g., over the phone) and accordingly, the auditory system can parse incoming speech information with high-temporal acuity (Poeppel, 2003; Morillon et al., 2010; Giraud and Poeppel, 2012). Conversely, visual speech alone leads to poor intelligibility scores (Campbell, 1989; Massaro, 1998) and visual processing is characterized by a slower sampling rate (Busch and VanRullen, 2010). The slow timescales over which visible articulatory gestures evolve (and are extracted by the observer's brain) constrain the representational granularity of visual information to visemes, categories much less distinctive than phonemes.

In auditory neuroscience, the specificity of phonetic processing and phonological categorization has long been investigated (Maiste et al., 1995; Simos et al., 1998; Liégeois et al., 1999; Sharma and Dorman, 1999; Philips et al., 2000). The peripheral mammalian auditory system has been proposed to efficiently encode a broad category of natural acoustic signals by using a time-frequency representation (Lewicki, 2002; Smith and Lewicki, 2006). In this body of work, the characteristics of auditory filters heavily depend on the statistical characteristics of sounds: as such, auditory neural coding schemes show plasticity as a function of acoustic inputs. The intrinsic neural tuning properties allow for multiple modes of acoustic processing with trade-offs in the time and frequency domains which naturally partition the time-frequency space into sub-regions. Complementary findings show that efficient coding can be realized for speech inputs (Smith and Lewicki, 2006) supporting the notion that the statistical properties of auditory speech can drive different modes of information extraction in the same neural populations, an observation supporting the “speech mode” hypothesis (Remez et al., 1998; Tuomainen et al., 2005; Stekelenburg and Vroomen, 2012).

In visual speech, how the brain derives speech-relevant information from seeing the dynamics of the facial articulators remains unclear. While the neuropsychology of lipreading has been thoroughly described (Campbell, 1986, 1989, 1992), very few studies have specifically addressed the neural underpinnings of visual speech processing (Calvert, 1997; Calvert and Campbell, 2003). Visual speech is a particular form of biological motion which readily engages some face-specific sub-processes (Campbell, 1986, 1992) but remains functionally independent from typical face processing modules (Campbell, 1992). Insights on the neural bases of visual speech processing may be provided by studies of biological motion (Grossman et al., 2000; Vaina et al., 2001; Servos et al., 2002) and the finding of mouth-movement specific cells in temporal cortex provides a complementary departing point (Desimone and Gross, 1979; Puce et al., 1998; Hans-Otto, 2001). Additionally, case studies (sp. prosopagnosia and akinetopsia) have suggested that both form and motion are necessary for the processing of visual and AV speech (Campbell et al., 1990; Campbell, 1992). In line with this, an unexplored hypothesis for the neural encoding of facial kinematics is the use form-from-motion computations (Cathiard and Abry, 2007) which could help the implicit recovery of articulatory commands from seeing the speaking face (e.g., Viviani et al., 2011).

### Active sampling of visual speech cues

In spite of the limited informational content provided by visual speech (most articulatory gestures remain hidden), AV speech integration is resilient to further degradation of the visual speech signal. Numerous filtering approaches do not suppress integration (Rosenblum and Saldaña, 1996; Campbell and Massaro, 1997; Jordan et al., 2000; MacDonald et al., 2000) suggesting that the use of multiple visual cues [e.g., luminance patterns (Jordan et al., 2000); kinematics (Rosenblum and Saldaña, 1996)]. Additionally, neither the gender (Walker et al., 1995) nor the familiarity (Rosenblum and Yakel, 2001) of the face impacts the robustness of AV speech integration. As will be discussed later, AV speech integration also remains resilient to large AV asynchronies (cf. *Resilient temporal integration and the co-modulation hypothesis*). Visual kinematics alone are sufficient to maintain a high rate of AV integration (Rosenblum and Saldaña, 1996) but whether foveal (i.e., explicit lip-reading with focus on the mouth area) or extra-foveal (e.g., global kinematics) information is most relevant for visemic categorization remains unclear.

Interestingly, gaze fixations 10–20° away from the mouth are sufficient to extract relevant speech information but numerous eye movements have also been reported (Vatikiotis-Bateson et al., 1998; Paré et al., 2003). It is noteworthy that changes of gaze direction can be crucial for the extraction of auditory information as neural tuning properties throughout the auditory pathway are modulated by gaze direction (Werner-Reiss et al., 2003) and auditory responses are affected by changes in visual fixations (Rajkai et al., 2008; van Wassenhove et al., 2012). These results suggest an interesting working hypothesis: the active scanning of a speaker's face may compensate for the slow sampling rate of the visual system.

Hence, despite the impoverished signals provided by visual speech, additional degradation does not fully prevent AV speech integration. As such, (supramodal) AV speech processing is more likely than not a natural mode of processing in which the contribution of visual speech to the perceptual outcome may be regulated as a function of the needs for perceptual completion in the system.

### AV speech mode hypothesis

Several findings have suggested that AV signals displayed in a speech vs. a non-speech mode influence both behavioral and electrophysiological responses (Tuomainen et al., 2005; Stekelenburg and Vroomen, 2012). Several observations could complement this view. First, lip-reading stands as a natural ability that is difficult to improve (as opposed to reading ability; Campbell, 1992) and is a good predictor of AV speech integration (Grant et al., 1998). In line with these observations, and as will be discussed later on, AV speech integration undergoes a critical acquisition period (Schorr et al., 2005).

Second, within the context of an internal speech model, AV speech integration is not arbitrary and follows principled internal rules. In the seminal work of McGurk and MacDonald (1976, MacDonald and McGurk, 1978), two types of phenomena illustrate principled ways in which AV speech integration occurs. In *fusion*, dubbing an auditory bilabial (e.g., [ba] or [pa]) onto a visual velar place of articulation (e.g., [ga] or [ka]) leads to an illusory fused alveolar percept (e.g., [da] or [ta], respectively). Conversely, in *combination*, dubbing an auditory [ga] onto a visual place of articulation [ba] leads to the illusory combination percept [bga]. Fusion has been used as an index of automatic AV speech integration because it leads to a unique perceptual outcome that is nothing like any of the original sensory inputs (i.e., neither a [ga] nor a [ba], but a third percept). Combination has been much less studied: unlike fusion, the resulting percept is not unique but rather a product of co-articulated speech information (such as [bga]). Both fusion and combination provide convenient (albeit arguable) indices on whether AV speech integration has occurred or not. These effects can be generalized across places-of-articulation in stop-consonants such that any auditory bilabial dubbed onto a visual velar result in a misperceived alveolar. These two kinds of illusory AV speech outputs illustrate the complexity of AV interactions and suggest that the informational content carried by each sensory modality determines the nature of AV interactions during speech processing. A strong hypothesis is that internal principles should depend on the articulatory repertoire of a given language and few cross-linguistic studies have addressed this issue (Sekiyama and Tohkura, 1991; Sekiyama, 1994, 1997).

Inherent to the speech mode hypothesis is the attentional-independence of speech analysis. Automaticity in AV speech processing (and in multisensory integration) is a matter of great debate (Talsma et al., 2010). A recent finding (Alsius and Munhall, 2013) suggests that conscious awareness of a face is not necessary for McGurk effects (cf. also Vidal et al. submitted, pers. communication). While attention may regulate the weight of sensory information being processed in each sensory modality—e.g., via selective attention (Lakatos et al., 2008; Schroeder and Lakatos, 2009)—attention does not a priori overtake the internal generative rules for speech processing. In other words, while the strength of AV speech integration can be modulated (Tiippana et al., 2003; Soto-Faraco et al., 2004; Alsius et al., 2005; van Wassenhove et al., 2005), AV speech integration is not fully abolished in integrators.

The robustness and principled ways in which visual speech influences auditory speech processing suggest that the neural underpinnings of AV speech integration rely on specific computational mechanisms that are constrained by the internal rules of the speech processing system—and possibly modulated by attentional focus on one or the other streams of information. I now elaborate on possible predictive implementations and tenants of AV speech integration.

## Predictive coding, priors and the bayesian brain

A majority of mental operations are cognitively impenetrable i.e., inaccessible to conscious awareness (Pylyshyn, 1984; Kihlstrom, 1987). Proposed more than a century ago [Parrot (cf. Allik and Konstabel, 2005); Helmholtz MacKay, 1958; Barlow, 1990; Wundt (1874)], unconscious inferences later coined the role of sensory processing as a means to remove redundant information in the incoming signals based on the informed natural statistics of sensory events. For instance, efficient coding disambiguates incoming sensory information using mutual inhibition as a means to decorrelate mixed signals: a network can locally generate hypotheses on the basis of a known (learned) matrix from which inversion can be drawn for prediction (Barlow, 1961; Srinivasan et al., 1982; Barlow and Földiak, 1989). Predictive coding can be local, for instance with a specific instantiation in the architecture of the retina (Hosoya et al., 2005). Early predictive models have essentially focused on the removal of redundant information in the spatial domain. Recently, predictive models have incorporated more sophisticated levels of predictions (Harth et al., 1987; Rao and Ballard, 1999; Friston, 2005). For instance, Harth et al. (1987) proposed a predictive model in which feedback connectivity shapes the extraction of information early in the visual hierarchy and such regulation of V1 activity in the analysis of sensory inputs has also been tested (Sharma et al., 2003). The initial conception of “top–down” regulation has been complemented with the notion that feed-forward connections may not carry the extracted information *per se* but rather the residual error between “top–down” internal predictions and the incoming sensory evidence (Rao and Ballard, 1999).

A growing body of evidence supports the view that the brain is a hierarchically organized inferential system in which internal hypotheses or predictions are generated at higher levels and tested against evidence at lower levels along the neural pathways (Friston, 2005): predictions are carried by backward and lateral connections whereas prediction errors are carried by forward projections. Predictive coding schemes have thus gone from local circuitries to brain system seemingly suggesting that access to high-level representations are necessary to formulate efficient predictions.

### Fixed vs. informed priors

Conservatively, any architectural constraint (e.g., connectivity pattern, gross neuroanatomical pathways), knowledge and circuitry acquired during a sensitive and before a critical period, or the endowment of the system can all be considered deterministic or *fixed priors*. Contrariwise, *informed priors* are any form of knowledge undergoing updates available through plastic changes and acquired through experience.

At the system level, a common neurophysiological index taken as evidence for predictive coding in cortex is the MisMatch Negativity (MMN) response (Näätänen et al., 1978; Näätänen, 1995): the MMN is classically elicited by the presentation of a rare event (~20% of the time) in the context of standard events (~80% of the time). The most convincing evidence for the MMN as a residual error resulting from the comparison of an internal prediction with incoming sensory evidence is the case of the MMN to omission, namely an MMN elicited when an event is omitted in a predictable sequence of events (Tervaniemi et al., 1994; Yabe et al., 1997; Czigler et al., 2006). Other classes of electrophysiological responses have been interpreted as residual errors elicited by a deviance at different levels of perceptual or linguistic complexities (e.g., the N400; Lau et al., 2008). Recent findings have also pointed out to the hierarchical level at which statistical contingencies can be incorporated in a predictive model (Wacongne et al., 2011). Altogether, these results are in line with recent hierarchical processing of predictive coding in which the complexity of the prediction depends on the depth of recursion in the predictive model (Kiebel et al., 2008).

In AV speech, the seminal work of Sams and Aulanko (1991) used an MMN paradigm with magnetoencephalography (MEG). Using congruent and incongruent (McGurk: audio [pa] dubbed onto visual [ka]) stimuli, the authors found that the presentation of an incongruent (congruent) AV speech deviant in a stream of congruent (incongruent) AV speech standards elicited a robust auditory MMN. Since, a series of subsequent MMN studies has replicated these findings (Colin et al., 2002; Möttönen et al., 2002, 2004) and the sources of the MMN was consistently located in auditory association areas, about 150 to 200 ms following auditory onset and in the superior temporal sulcus from 250 ms on. The bulk of literature using MMN in AV speech therefore suggests that internal predictions generated in the auditory regions incorporate visual information relevant for the analysis of speech.

Critically, it is here argued that internal models invoked for speech processing are part of the cognitive architecture i.e., likely endowed with fixed priors for the analysis of (speech) inputs. The benefit of positing an internal model is precisely to account for robust and invariant internal representations that are resilient to the ever-changing fluctuations of a sensory environment. As such, a predictive model should help refine the internal representations in light of sensory evidence, not entirely shape the internal prediction on the basis of the temporary environmental statistics.

In this context, the temporal statistics of stimuli using an MMN paradigm (e.g., 80% standards, 20% deviants) confine predictions to the temporary experimental context: the residual error is context-specific and tied to the temporary statistics of inputs provided within a particular experimental session. Thus, the MMN may not necessarily reveal fixed priors or specific hard-wired constrains of the system. An internal model should provide a means to stabilize non-invariance in order to counteract the highly variable nature of speech utterances irrespective of the temporally local context. A strong prediction is thus that the fixed priors of an internal model should supersede the temporary statistics of stimuli during a particular experimental session. Specifically, if predictive coding is a canonical operation of cortical function, residual errors should be the rule, not the exception and residual errors should be informative with respect to the content of the prediction, not only with respect to the temporal statistics of the sensory evidence. Following this observation, an experimental design using an equal number of different types of stimuli should reveal predictive coding indices that specifically target the hard-constraints or fixed priors of the system. In AV speech, auditory event-related potentials elicited by the presentation of AV speech stimuli show dependencies on the content of visual speech stimuli: auditory event-related potentials could thus be interpreted as the resulting residual-errors of a comparison process between auditory and visual speech inputs (van Wassenhove et al., 2005).

The argument elaborated here is that to enable a clear interpretation of neurophysiological and neuroimaging data using predictive approaches, the description of the internal model being tested along with the levels at which predictions are expected to occur (hence, the representational format and content of the internal predictors) has become necessary. For instance, previous electrophysiological indices of AV speech integration (van Wassenhove et al., 2005) including latency (interpreted as visual modulations of auditory responses that are speech content-dependent) and amplitude (interpreted as visual modulations of auditory responses that are speech content-independent) effects are not incompatible with the amplitude effects reported in other studies (e.g., Stekelenburg and Vroomen, 2007). AV speech integration implicates speech-specific predictions (e.g., phonetic, syllabic, articulatory representations) but also entails more general operations such as temporal expectation or attentional modulation. As such, the latency effects showed speech selectivity whereas amplitude effects did not; the former may index speech-content predictions coupled with temporal expectations, whereas the latter may inform on general predictive rules. Hierarchical levels can operate predictively in a non-exclusive and parallel manner. The benefit of predictive coding approaches is thus the refinement internal generative models, their specificity with regards to the combinatorial rules that are being used and the representational formats and contents of the different levels of predictions implicated in the model.

### Bayesian implementation of predictive coding

Can Bayesian computations serve predictive coding for speech processing? Recent advances in computational neurosciences have offered a wealth of insights on the Bayesian brain (Denève and Pouget, 2004; Ernst and Bülthoff, 2004; Ma et al., 2006; Yuille and Kersten, 2006) and have opened new and essential venues for the interpretation of perceptual and cognitive operations.

AV speech research has seen the emergence of one of the first Bayesian models for perception, the Fuzzy Logical Model of Perception or FLMP (Massaro, 1987, 1998). In the initial FLMP, the detection and the evaluation stages in speech processing were independent and eventually merged into a single evaluation process (Massaro, 1998). At this level, each speech signals is independently evaluated against prototypes in memory store and assigned a “fuzzy truth value” representing how well the input matches a given prototype. The fuzzy truth value could range from 0 (does not match at all) to 1 (exactly matches the prototype); the prototypical feature represents the ideal value that an exemplar of the prototype holds—i.e., 1 in fuzzy logic—hence the probability that a feature is present in the speech inputs. The prototypes are defined as speech categories which provide an ensemble of features and their conjunctions (Massaro, 1987). In AV speech processing, the 0 to 1 mapping in each sensory modality allowed the use of Bayesian conditional probabilities and computations would take the following form: what is the probability that an AV speech input is a [ba] given a 0.6 probability of being a bilabial in the auditory domain and a 0.7 probability in the visual domain? The best outcome is selected based on the goodness-of-fit determined by prior evidence through a maximum likelihood procedure. Hence, in this scheme, the independence of sensory modalities is necessary to allow the combination of two feature estimates (e.g., place-of-articulations) and a compromise is reached at the decision stage through adjustments of the model with additional sensory evidence. In the FLMP, phonological categorization is thus replaced by a syllabic-like stage (and word structuring) as constrained by the classic phonological rules.

A major criticism of this early Bayesian model for speech perception pertains to the fitting adjustments of the FLMP which would either overfit or be inappropriate for the purpose of predicting integration (Grant, 2002; Schwartz, 2003). Additional discussions have pointed out to the lack of clear accounting of the format of auditory and visual speech representations in such models (Altieri et al., 2011). More recent proposals have notably proposed a parallel architecture to account for AV speech integration efficiency in line with the interplay of inhibitory and excitatory effects seen in neuroimaging data (Altieri and Townsend, 2011).

## Analysis-by-synthesis (AbyS)

**Figure 2:**
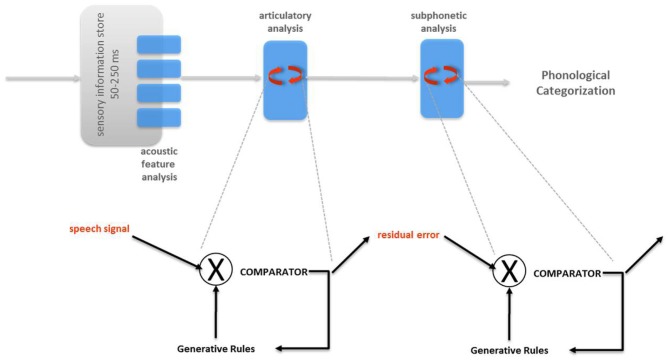
**Analysis-by-synthesis (Halle and Stevens, 1962)**. In the original proposal, two major successive predictive modules are postulated: articulatory analysis followed by subphonetic analysis. In both modules, the generative rules of speech production are used to emit and refine predictions of the incoming sensory signal (articulatory analysis) or residual error from the previous stage (subphonetic analysis).

In the seminal description of Analysis-by-Synthesis (AbyS, Figure 2) for auditory speech processing by Halle and Stevens (1962), and in line with the Motor Theory of Speech Perception (Liberman et al., 1967; Liberman and Mattingly, 1985), the internal representations used for the production and perception of speech are shared. Specifically, AbyS sketched a predictive implementation for the analysis of auditory speech: the internalized rules for speech production enable to generate hypotheses about which acoustic inputs would come next (Stevens, 1960). From a computational standpoint, AbyS provides the representational system and the fixed priors (internal rules) constraining the computations of Bayesian probabilities at the comparison stages. The comparison of auditory and visual speech inputs with internalized articulatory commands can be compatible with Bayesian computations.

In the AbyS, auditory inputs (after preliminary spectral analysis Poeppel et al., 2008) are matched against the internal articulatory rules that would be used to produce the utterance (Halle and Stevens, 1962). Internal speech production rules can take upon continuous values as the set of commands in speech production change as a function of time but “a given articulatory configuration may not be reached before the motion toward the next must be initiated” (Halle and Stevens, 1962). Although the internal rules provide a continuous evaluation of the parameters, the evaluation process can operate on a different temporal scale thereby the units of speech remain discrete and articulatory based. By analogy with the overlap of articulatory commands, the auditory speech inputs contain the traces of preceding and following context (namely, co-articulation effects). Hence, the continuous assignment of values need not bear a one-to-one relationship with the original input signals and overlapping streams of information extraction (for instance, via temporal encoding windows) may enable this process.

### Amodal predictions

This early model provided one possible implementation for a forward in time and predictive view of sensory analysis (Stevens, 1960; Halle and Stevens, 1962). Since, AbyS has been re-evaluated in light of recent evidence for predictive coding in speech perception (Poeppel et al., 2008). The internally generated hypotheses are constrained by phonological rules and their distinctive features serve as the discrete units for speech production/perception (Poeppel et al., 2008). The non-invariance of incoming speech inputs can be compensated for by the existence of trading cues matched against the invariant built-in internal rules of the speech system. In particular, the outcome of the comparison process (i.e., the residual error) enables an active correction of the perceptual outcome (i.e., recalibrating so as to match the best fitting value) of the production output.

**Figure 3:**
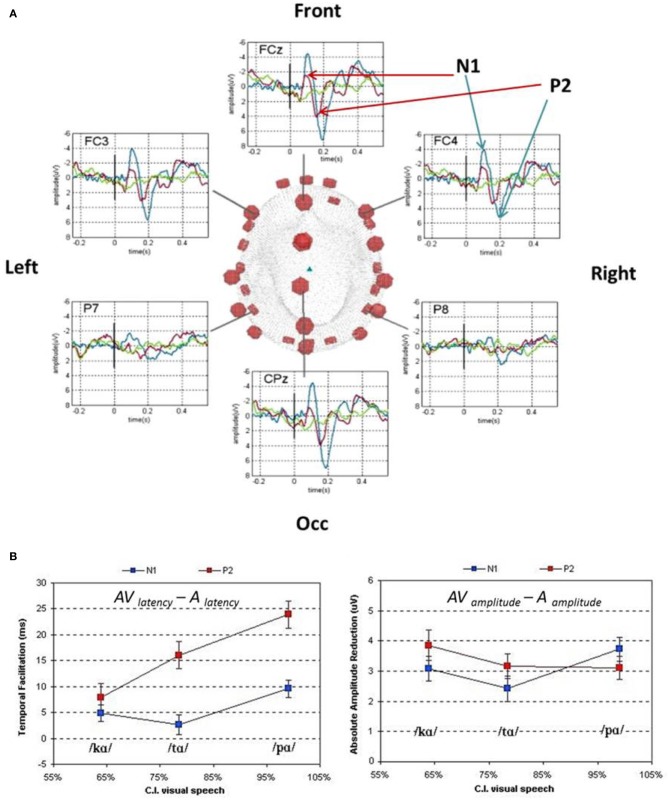
**Auditory event-related potentials in response to auditory (blue), visual (green), and AV (red) non-sense syllables**. (Panel **A**) Scalp distribution of auditory ERPs to auditory, visual and AV speech presentation. (Panel **B**) Latency (bottom left) and absolute amplitude (bottom right) differences of the auditory ERPs (N1 is blue, P2 is red) as a function of correct identification (CI) of visual speech. The better the identification rate in visual speech alone, the earlier the N1/P2 complex occurred. A similar amplitude decrease for N1 (less negative) and P2 (less positive) was observed for all congruent and incongruent AV presentations as compared to A presentations (van Wassenhove et al., 2005).

In conversational settings, the visible articulatory gestures for speech production have recently been argued to precede the auditory utterance by an average of 100–300 ms (Chandrasekaran et al., 2009). The natural precedence of visual speech features could initiate the generation of internal hypotheses as to the incoming auditory speech inputs. This working hypothesis was tested with EEG and MEG by comparing the auditory evoked-responses elicited by auditory and AV speech stimuli (van Wassenhove et al., 2005; Figure 3). The early auditory evoked responses elicited by AV speech showed (i) shorter latencies and (ii) reduced amplitudes compared to those elicited by auditory speech alone (van Wassenhove et al., 2005; Arnal et al., 2009). Crucially, the latency shortening of auditory evoked responses was a function of the ease with which participants categorized visual speech alone, thereby a [pa] lead to shorter latencies than [ka] or [ta]. In the context of AbyS, the reliability with which visual speech can trigger internal predictions for incoming auditory speech constrains the analysis of auditory speech (van Wassenhove et al., 2005; Poeppel et al., 2008; Arnal et al., 2009, 2011).

### Temporal encoding windows and temporal windows of integration

**Figure 4:**
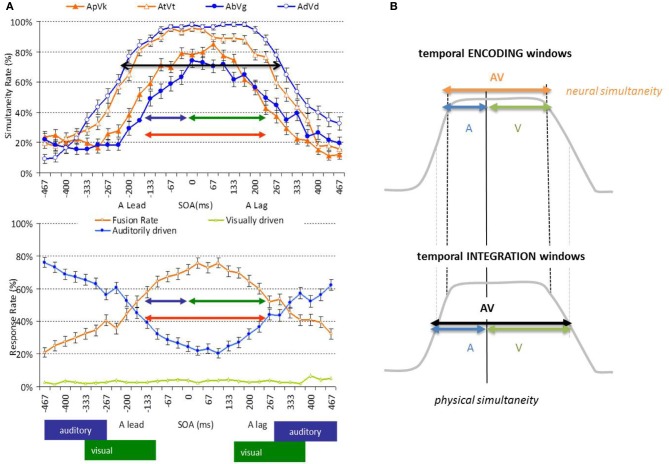
**Temporal window of integration in AV speech**. (Panel **A**) Illustration of results in a simultaneity judgment task (top) and a speech identification task (bottom) (van Wassenhove et al., 2007). Simultaneity ratings observed for congruent (top, filled symbols) and incongruent (top, open symbols) AV speech as a function of AV desynchrony. Auditory dominated (bottom, blue), visual dominated (bottom, green) or McGurk fusion (bottom, orange) responses as a function of desynchrony using McGurked syllables. The combination of the auditory encoding (blue arrow: tolerance to visual lags) and visual encoding (green arrow: tolerance to visual leads) form the temporal encoding window for AV speech integration. (Panel **B**) Schematic illustration distinguishing temporal encoding and temporal integration windows. The temporal resolution reflected in the encoding window corresponds to the necessary or obligatory time for speech encoding; the temporal resolution reflected in the integration windows correspond to the encoding window plus the tolerated temporal noise leading to less than optimal encoding performance.

**Figure 5:**
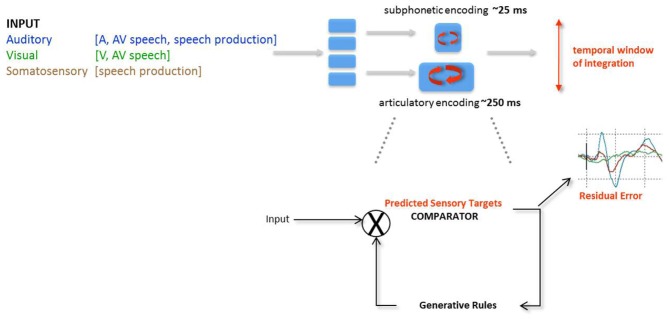
**Analysis-by-synthesis (AbyS) in AV speech processing**. Two analytical routes are posited on the basis of the original AbyS proposal, namely a subphonetic feature and an articulatory analysis of incoming speech inputs. The privileged route for auditory processing is subphonetic by virtue of the fine temporal precision afforded by the auditory system; the privileged route for visual speech analysis is articulatory by virtue of slower temporal resolution of the visual system and the kinds of information provided by the interlocutor's face. Evidence for the coexistence of 2 modes of speech processing or temporal multiplexing of AV speech can be drawn from the asymmetry of the temporal window of integration in AV speech (cf. Figure 4). Although both stages are posited to run in parallel, predictions in both streams are elaborated on the basis of the generative rules of speech production. Predictive mode of AV speech processing is notably marked by a decreased amplitude of the auditory evoked responses (van Wassenhove et al., 2005; Arnal et al., 2009) and residual errors have been characterized either by latency shifts of the auditory evoked responses commensurate with the gain of information in visual speech (van Wassenhove et al., 2005) or by later amplitudes differences commensurate to the detected incongruency of auditory and visual speech inputs (Arnal et al., 2009). AbyS is thus a predictive model operating on temporal multiplexing of speech (i.e., parallel and predictive processing of speech features on two temporal scales) and is compatible with recently proposed neurophysiological implementations of predictive speech coding (Poeppel, 2003; Giraud and Poeppel, 2012).

Two features of the AbyS model are of particular interest here (Figure 5). First, visual speech is argued to predict auditory speech in part because of the natural precedence of incoming visual speech inputs; second, AV speech integration tolerates large AV asynchronies without affecting optimal integration (Massaro et al., 1996; Conrey and Pisoni, 2006; van Wassenhove et al., 2007; Maier et al., 2011). In one of these studies (van Wassenhove et al., 2007), two sets of AV speech stimuli (voiced and voiceless auditory bilabials dubbed onto visual velars) were desynchronized and tested using two types of task: (i) a speech identification task (“what do you hear while looking at the talking face?”) and (ii) a temporal synchrony judgment task (“where AV stimuli in- or out-of-sync?). Results showed that both AV speech identification and temporal judgment tolerated about 250 ms of AV desynchrony in McGurked and congruent syllables. The duration of the “temporal window of integration” found in these experiments approximated the average syllabic duration across languages, suggesting that syllables may be an important unit of computations in AV speech processing. Additionally, this temporal window of integration showed an asymmetry so that visual leads were better tolerated than auditory leads—with respect to the strength of AV integration. This suggested that the temporal resolutions for the processing of speech information arriving in each sensory modality may actually differ, in agreement with the natural sampling strategies found in auditory and visual systems. This interpretation could now be refined (Figure 4).

The “temporal window of integration” can be seen as the integration of two temporal encoding windows (following the precise specifications of Theunissen and Miller, 1995), namely: the encoding window needed by the auditory system to reach phonological categorization is determined by the tolerance to visual speech lags, whereas the encoding window needed for the visual system to reach visemic categorization is illustrated by the tolerance to auditory speech lags. Hence, the original “temporal window of integration” is a misnomer: the original report describing a plateau within which the order of auditory and speech information did not diminish the rate of integration specifically illustrates the “temporal encoding window” of AV speech i.e., *the necessary time needed for the speech system to elaborate a final outcome or to establish a robust residual error from the two analytical streams in the AbyS framework*. The tolerated asynchronies measured by just-noticeable-differences (Vroomen and Keetels, 2010) or thresholds should be interpreted as the actual “temporal integration window” namely, the tolerance to temporal noise in the integrative system. Said differently, *the fixed constraints are the temporal encoding windows; the tolerance to noise is reflected in the temporal integration windows*.

Temporal windows of integration or “temporal binding windows” (Stevenson et al., 2012) have been observed for various AV stimuli and prompted some promising models for the integration of multisensory information (Colonius and Diederich, 2004). Consistent with the distinction between encoding and integration windows described above, a refined precision of temporal integration/binding windows can be obtained after training (Powers et al., 2009) with a likely limitation of training to the temporal encoding resolution of the system. Interestingly, a recent study (Stevenson et al., 2012) has shown that the width of an individual's temporal integration window for non-speech stimuli could predict the strength of AV speech integration (Stevenson et al., 2012). Whether direct inferences can be drawn between the conscious simultaneity of AV events (overt comparison of events timing entails segregation) and AV speech (integration of AV speech content) is, however, growing controversial. For instance, temporal windows in patients with schizophrenia obtained in a timing task are a poor predictors of their ability to bind AV speech information (Martin et al., 2012), suggesting that distinct neural processes are implicated in the two tasks (in spite of identical AV speech stimuli). Future work in the field will likely help disambiguating which neural operations are sufficient and necessary for conscious timing and which are necessary for binding operations.

### Oscillations and temporal windows

In this context, one could question whether the precedence of visual speech is a prerequisite for predictive coding in AV speech and specifically, whether the ordering of speech inputs in each sensory modality may affect the posited predictive scheme. This would certainly be an issue if speech analysis followed serial computations operating on a very refined temporal grain. As seen in studies of desynchronized AV speech, this does not seem to be the case: the integrative system operates on temporal windows within which order is not essential (cf. van Wassenhove, 2009 for a discussion on this topic) and both auditory and visual systems likely use different sampling rates in their acquisition of sensory evidence (cf. Temporal parsing and non-invariance).

Recent models of speech processing have formulated clear mechanistic hypotheses implicating neural oscillations: the temporal logistics of cortical activity naturally impose temporal granularities on the parsing and the integration of speech information (Giraud and Poeppel, 2012). For instance, the default oscillatory activity observed in the speech network (Morillon et al., 2010) is consistent with the posited temporal multiplexing of speech inputs. If the oscillatory hypothesis is on the right track, it is thus very unlikely that the dynamic constraints as measured by the temporal encoding (and not integration) window can be changed considering that cortical rhythms (Wang, 2010) provide the dynamic architecture for neural operations. The role of oscillations for predictive operations in cortex has further been reviewed elsewhere (Arnal and Giraud, 2012).

Additionally, visual speech may confer a natural rhythmicity to the syllabic parsing of auditory speech information (Schroeder et al., 2008; Giraud and Poeppel, 2012) and this could be accounted for by phase-resetting mechanisms across sensory modalities. Accordingly, recent MEG work illustrates phase consistencies during the presentation of AV information (Luo et al., 2010; Zion Golumbic et al., 2013). Several relevant oscillatory regimes [namely theta (4 Hz, ~250 ms), beta (~20 Hz, 50 ms) and gamma (>40 Hz, 25 ms)] have also been reported that may constrain the integration of AV speech (Arnal et al., 2011). A bulk of recent findings provides structuring constraints on speech processing—i.e., fixed priors. Consistent with neurophysiology, AbyS incorporates temporal multiplexing for speech processing thereby parallel temporal resolutions are used to represent relevant speech information at the segmental and syllabic scales (Poeppel, 2003; Poeppel et al., 2008). In AV speech, each sensory modality may thus operate with a preferred temporal granularity and it is the integration of the two processing streams that effectively reflects the temporal encoding window. Such parallel encoding may also be compatible with recent efforts in modeling AV speech integration (Altieri and Townsend, 2011).

## Critical period in AV speech perception: acquisition of fixed priors

During development, the acquisition of speech production could undergo an imitative stage from visual speech perception to speech production. In principle, the imitative stage allows children to learn how to articulate speech sounds by explicitly reproducing the caretakers' facial gestures. However, mounting evidence suggests that imitation does not operate on a blank-slate system; rather, internal motor representations for speech are readily available early on. First, the gestural repertoire is already very rich only 3 weeks after birth, suggesting an innate ability for the articulation of elementary speech sounds (Meltzoff and Moore, 1979; Dehaene-Lambertz and DehaeneHertz-Pannier, 2002). Second, auditory inputs alone are sufficient for infants to reproduce accurately simple speech sounds and enable the recognition of visual speech inputs matching utterances that have only been heard (Kuhl and Meltzoff, 1982, 1984). Furthermore, during speech acquisition, infants do not see their own gestures: consequently, infants can only correct their own speech production via auditory feedback or via matching a peer's gestures (provided visually) to their own production, i.e., via proprioception (Meltzoff, 1999).

Comparatively few studies have addressed the question of AV speech processing during development. The simplest detection of AV synchrony has been argued to emerge first followed by duration, rate and rhythm matching across sensory modalities in the first 10 months of an infant's life (Lewkowicz, 2000). In the spatial domain, multisensory associations are established slowly during the first 2 years of life suggesting that the more complex the pattern, the later the acquisition, in agreement with the “increasing specificity hypothesis” (Gibson, 1969; Spelke, 1981). Three and a half months old infants are sensitive to natural temporal structures but only later on (7 months) are arbitrary multisensory associations detected (e.g., pitch and shape Bahrick, 1992); emotion matching in strangers (Walker-Andrews, 1986). However, early sensitivity to complex AV speech events has been reported in 5 months old infants who can detect the congruency of auditory speech inputs with facial articulatory movements (Rosenblum et al., 1997). The spatiotemporal structuring of arbitrary patterns as well as the nature and ecological relevance of incoming information owe to be important factors in the tuning of a supramodal system. The acquisition of cross-sensory equivalences seems to undergo a perceptual restructuring that can be seen as a fine-tuning of perceptual grouping (Gestalt-like) rules.

Born deaf children who received implants at various ages provide an opportunity to investigate the importance of age at the time of implant for the development of AV speech perception (Bergeson and Pisoni, 2004). A substantial proportion of children who receive cochlear implants learn to perceive speech remarkably well using their implants (Waltzman et al., 1997; Svirsky et al., 2000; Balkany et al., 2002) and are able to integrate congruent AV speech stimuli (Bergeson et al., 2003, 2005; Niparko et al., 2010). In a previous study (Schorr et al., 2005), born-deaf children who had received cochlear implants were tested with McGurk stimuli [visual [ka] dubbed with auditory [pa]; (McGurk and MacDonald, 1976)]. The main hypothesis was that experience played a critical role in forming AV associations for speech perception. In this study, most children with cochlear implants did not experience reliable McGurk effects, and AV speech perception for these children was essentially dominated by lip-reading consistent with their hearing-impairment. However, the likelihood of consistent McGurk illusory reports depended on the age at which children received their cochlear implants. Children who exhibited consistent McGurk illusions received their implants before 30 months of age; conversely, children who received implants after 30 months of age did not show consistent McGurk effects. These results demonstrated that AV speech integration was shaped by experience early on in life. When auditory experience with speech was mediated by a cochlear implant, the likelihood of acquiring strong AV speech fusion was greatly increased. These results suggested the existence of a sensitive period for AV speech perception (Sharma et al., 2002).

To date however, whether the temporal constraints and neurophysiological indices for AV speech integration in development are comparable to those observed in adults remain unclear.

## Resilient temporal integration and the co-modulation hypothesis

In natural scenes, diverse sensory cues help the brain select and integrate relevant information to build internal representations. In the context of perceptual invariance and supramodal processing, auditory pitch and visual spatial frequency have been shown to undergo automatic cross-sensory matching (Maeda et al., 2004; Evans and Treisman, 2010). Additionally, auditory and visual signals showing slow temporal fluctuations are most likely to undergo automatic integration (Kösem and van Wassenhove, 2012). In AV speech, the acoustic envelope and the movements of the lips show high correlation or co-modulation (Grant and Seitz, 2000; Remez, 2003) naturally locked to the articulatory gestures of the face. Crucially, this co-modulation shows specificity: AV speech intelligibility shows a similar range of tolerance to asynchronies when the spectral characteristics of the acoustic signal preserve the feature information specific to the articulation (i.e., the F2/F3 formants region) (Grant and Greenberg, 2001). These local correlations have recently been argued to promote AV speech integration even when visual speech information is consciously suppressed (Alsius and Munhall, 2013). Taken altogether, these results suggest that the correlation of auditory and visual speech signals serve as a strong (bottom-up) cue for integration enabling the brain to correctly track signals belonging to the same person as indicated by recent neurophysiological findings (Zion Golumbic et al., 2013).

These observations need to be reconciled with an efficient predictive coding framework as the speech content provided by audition and vision is likely undergoing a non-correlative operation. This would be necessary to allow for the typical informational gain observed in AV speech studies in line with a previously sketched out idea (van Wassenhove et al., 2005), the proposed distinction between correlated and complementary modes of AV speech processing (Campbell, 2008) and AV speech integration models (Altieri and Townsend, 2011).

In this context, while there is ample evidence that speaking rate has a substantial impact on AV speech perception, little is known about the effect of speaking rate on the temporal encoding window. Changes in speaking rate naturally impact the kinematics of speech production, hence the acoustic and visual properties of speech. It is unclear to which extent the posited hard temporal constraints on AV speech integration may be flexible under various speaking rates. In the facial kinematics, different kinds of cues can effectively vary including the motion of the surface structures, the velocity patterns of the articulators and the frequency components over a wide spectrum. Any or all of these could contribute differently to AV speech integration for fast and slow speech and could thus perturb the integration process.

In two experiments (Brungart et al., 2007, 2008), the resilience of AV speech intelligibility was put to the test of noise, AV speech asynchrony and speaking rate. In a first experiment, AV speech recordings of phrases from the Modified Rhyme Test (MRT) were accelerated or decelerated (Brungart et al., 2007). Eight different levels of speaking rate were tested ranging from 0.6 to 20 syllables per second (syl/s). Results showed that the benefits of AV speech were preserved at speaking rates as fast as 12.5 syl/s but disappeared when the rate was increased to 20 syl/s. Importantly, AV speech performance did not benefit from phrases presented slower than their original speaking rates. Using the same experimental material, both the speaking rate and the degree of AV speech asynchrony were varied (Brungart et al., 2008). For the fastest speaking rates, maximal AV benefit occurred at slightly larger visual delay (150 ms) but there was no conclusive evidence suggesting that auditory speech delays for maximal benefit systematically changed with speaking rate. At the highest speaking rates, AV speech enhancement was maximal when the audio signal was delayed by ~150 ms relative to visual speech, and performance degraded relatively rapidly when the audio speech varied away from its optimal value. As the speaking rate decreased, the range of delays for enhanced AV speech benefit increased, suggesting that participants were tolerant to a wider range of AV speech asynchronies when the speaking rate was relatively slow. However, there was no compelling evidence suggesting that the optimal delay value for AV enhancement systematically changed with the speaking rate of the talker. Finally, when acoustic noise was added, the benefit of visual cues degraded rapidly with faster speaking rate. AV speech integration in noise occurred at all speaking rates slower than 7.8 syl/s. AV speech benefits were observed in all conditions suggesting that the co-modulation of AV speech information can robustly drives integration.

## Neural mechanisms for AV speech processing: convergence and divergence

Two reliable electrophysiological markers for AV speech integration are (i) an amplitude decrease (Besle et al., 2004; Jääskeläinen et al., 2004; van Wassenhove et al., 2005; Bernstein et al., 2008; Arnal et al., 2009; Piling, 2009) and (ii) latency shifts (van Wassenhove et al., 2005; Arnal et al., 2009) of the auditory evoked responses. Decreased amplitude of the auditory response to visual speech inputs was originally observed when participants were shown with a video of a face articulating the same or a different vowel sound 500 ms after the presentation of the face (Jääskeläinen et al., 2004). In this study, visual speech inputs were interpreted as leading to the adaptation of the subset of auditory neurons responsive to that feature. However, no difference in amplitude was observed when the visual stimuli were drawn from the same or from a different phonetic category, suggesting non-specific interactions of visual speech information with the early auditory analysis of speech. The amplitude reduction of the auditory evoked responses observed in EEG and MEG is supported by intracranial recordings (Reale et al., 2007; Besle et al., 2008). In particular, Besle et al. (2008) reported two kinds of AV interactions in the secondary auditory association cortices after the first influence of visual speech in this region: at the onset of the auditory syllable, the initial visual influence disappeared and the amplitude of the auditory response decreased compared to the auditory alone presentation. Similar amplitude reductions were observed to the presentation of AV syllables over the left lateral pSTG (Reale et al., 2007).

In all of these studies, the reported amplitude reduction spanned a couple hundreds of milliseconds, consistent with the implication of low frequency neural oscillations. In monkey neurophysiology, a decreased low-frequency power in auditory cortex has been reported in the context of AV communication (Kayser and Logothetis, 2009). Based on a set of neurophysiological recordings in monkeys, it was proposed that visual inputs change the excitability of auditory cortex by resetting the phase of ongoing oscillation (Schroeder et al., 2008); recent evidence using an AV cocktail party design (Zion Golumbic et al., 2013) support this hypothesis. Additional MEG findings suggest that the tracking of AV speech information may be dealt with by phase-coupling of auditory and visual cortices (Luo et al., 2010). In the context of a recent neurocomputational framework for speech processing (Giraud and Poeppel, 2012), visual speech would thus influence ongoing auditory activity so as to condition the analysis of auditory speech events. Whether this tracking is distinctive with regards to speech content is unclear. The decreased amplitude of auditory evoked responses may be related to the phase entrainment between auditory and visual speech or to the power decrease of low-frequency regions. However, since no clear correlation between the amplitude and the phonetic content are seen in the amplitude, this mechanism does not appear to carry the content of the speech representation, consistent with the lack of visemic or AV speech congruency effect (van Wassenhove et al., 2005; Arnal et al., 2009) and a previously emitted interpretation (Arnal et al., 2009, 2011).

With respect to latency shifts, two studies reported auditory evoked responses as a function of visemic information: one study interpreted that effects on auditory evoked responses carried the residual error (van Wassenhove et al., 2005) and another reported late residual errors at about 400 ms (Arnal et al., 2009). The specificity of this modulation remains unsettled: visual inputs have been reported to change the excitability of auditory cortex by resetting the phase of ongoing oscillation (Lakatos et al., 2008) but an amplification of the signal would have been predicted in auditory cortex (Schroeder et al., 2008). A recent study (Zion Golumbic et al., 2013) implicates the role of attention in selecting or predicting relevant auditory inputs on the basis of visual information. This interpretation would be in line with the notion that visual speech information enables to increase the salience of relevant auditory information for further processing. To which extent phase-resetting mechanisms are speech-specific or more generally implicated in modulating the gain of sensory inputs remains to be determined, along with the implication of specific frequency regimes. Recent findings suggest that multiplexing of speech features could be accomplished in different frequency regimes (Arnal et al., 2011) with coupling between auditory and visual cortices realized via STS. The directionality of these interactions remains to be thoroughly described in order to understand how specific the informational content propagates in the connectivity of these regions. Recent work in monkey neurophysiology has started addressing these issues (Kayser et al., 2010; Panzeri et al., 2010).

It is noteworthy that MEG, EEG, and surface EEG (sEEG) data can contrast with fMRI and PET findings in which enhanced and supra-additive BOLD activations have been reported to the presentation of visual and AV speech. Both enhanced and sub-additive activation in mSTG, pSTG and pSTS have been reported together with left inferior temporal gyrus (BA 44/45), premotor cortex (BA 6), and anterior cingulate gyrus (BA 32) to the presentation of congruent and incongruent AV speech, respectively (Calvert, 1997; Calvert et al., 1999, 2000; Hasson et al., 2007; Skipper et al., 2007). Other fMRI findings (Callan et al., 2003) have shown significant activation of the MTG, STS, and STG in response to the presentation of AV speech in noise; BOLD activation consistent with the inverse effectiveness principle in these same regions (MTG, STS, and STG) has also been reported for stimuli providing information on the place of articulation (Callan et al., 2004). The left posterior STS has been shown sensitivity to incongruent AV speech (Calvert et al., 2000; Wright et al., 2003; Miller and D'Esposito, 2005). Using fMRI and PET, Sekiyama et al. (2003) used the McGurk effect with two levels of auditory noise; comparison between the low and high SNR conditions revealed a left lateralized activation in the posterior STS and BA 22, thalamus, and cerebellum. However, not all studies support the inverse effectiveness principle in auditory cortex (Calvert et al., 1999; Jones and Callan, 2003). Desynchronizing AV McGurk syllables does not significantly affect activation of the STS or auditory cortex (Olson et al., 2002; Jones and Callan, 2003) whereas others report significant and systematic activation of HG as a function of desynchrony (Miller and D'Esposito, 2005). Recent fMRI studies have reported specialized neural populations in the Superior Temporal Sulcus (STS in monkey) or Superior Temporal Cortex (STC, human homolog). The organization of this multisensory region is known to be patchy (Beauchamp et al., 2004) but recognized to be an essential part of the AV speech integration network (Arnal et al., 2009; Beauchamp et al., 2010). The middle STC (mSTC) is a prime area for the detection of AV asynchrony and the integration of AV speech (Bushara et al., 2001; Miller and D'Esposito, 2005; Stevenson et al., 2010, 2011). At least two neural subpopulations may coexist in this region: the synchrony population tagged S-mSTC showing increased activation to AV speech stimuli when the auditory and visual streams are in synchrony and the bimodal population tagged B-mSTC showing the opposite pattern, namely a decrease of activation with the presentation of synchronized audiovisual speech streams (Stevenson et al., 2010, 2011). These results may help shed light on the contribution of neural subpopulations in mSTC in computing redundant information vs. efficient coding for AV speech processing.

Using fMRI technique, the contribution of motor cortices has also been tested in the perception of auditory, visual and AV speech (Skipper et al., 2007). In these experiments, participants actively produced syllables or passively perceived auditory, visual and AV stimuli in the scanner. The AV stimuli consisted of both congruent AV [pa], [ka], and [ta] and McGurk fusion stimuli (audio [pa] dubbed onto a face articulating [ka]). The main results showed that the cortical activation pattern during the perception of visual and AV but not auditory speech greatly overlapped with that observed in speech production. The areas showing above 50% of overlap in production and perception were bilateral anterior and posterior Superior Temporal cortices (STa and STp, respectively), and ventral premotor cortex (PMv). The perception of McGurk fusion elicited patterns of activation that correlated differently across cortical areas with the perception of a congruent AV [pa] (the auditory component in the McGurk fusion stimulus), AV [ka] (the visual component of the McGurk fusion stimulus) or AV [ta] (the perceived illusory [ta] elicited by the McGurk fusion stimulus). Activations observed in frontal motor areas, and auditory and somatosensory cortices during McGurk presentation correlated more with the perceived syllable (AV [ta]) than the presented syllables in either sensory modality (A [pa], V [ka]). In visual cortices, activation correlated most with the presentation of a congruent AV [ka]. Overall, results were interpreted in the context of a forward model of speech processing.

## Outstanding questions

First, what is (in) a prediction? Although computational models provide interesting constraints with which to work, we cannot currently separate temporal prediction from speech-content predictions (e.g., Arnal and Giraud, 2012). One important finding encompassing the context of speech is that amplitude decrease can be seen as a general marker of predictive coding (e.g., Todorovic and de Lange, 2012) in auditory cortex and more specifically during speech production (Houde and Jordan, 1998).

Second, what anchors are used to parse visual speech information or, what are the “edges” (Giraud and Poeppel, 2012) of visual speech information? Complementarily, can we use cortical responses to derive the distinctive features of visual speech (Luo et al., 2010)?

Third, in the context of fixed temporal constraints for speech processing, how early can temporal encoding/integration windows be characterized in babies? Is the co-modulation hypothesis a general guiding principle for multisensory integration or a specific feature of AV speech?

Finally, the implication of the motor system in the analysis of speech inputs has been a long-standing debate undergoing increasing refinement (e.g., Scott et al., 2009). The inherent rhythmicity of speech production naturally constrains the acoustic and visual structure of auditory and visual speech outcomes. Is the primary encoding mode of AV speech articulatory or acoustic (e.g., Altieri et al., 2011; Schwartz et al., 2012)?

### Conflict of interest statement

The author declares that the research was conducted in the absence of any commercial or financial relationships that could be construed as a potential conflict of interest.

## References

[B1] Allik J. Konstabel K. (2005). G. F. Parrot and the theory of unconscious inferences. J. Hist. Behav. Sci. 41, 317–330 10.1002/jhbs.20114 16196051

[B2] Alsius A. Munhall K. G. (2013). Detection of audiovisual speech correspondences without visual awareness. Psychol. Sci. 24, 423–431 10.1177/0956797612457378 23462756

[B3] Alsius A. Navarra J. Campbell R. Soto-Faraco S. (2005). Audiovisual integration of speech falters under high attention demands. Curr. Biol. 15, 839–843 10.1016/j.cub.2005.03.046 15886102

[B4] Altieri N. Pisoni D. B. Townsend J. T. (2011). Some behavioral and neurobiological constraints on theories of audiovisual speech integration: a review and suggestions for new directions. Seeing Perceiving 24, 513–539 10.1163/187847611X595864 21968081 PMC3293210

[B5] Altieri N. Townsend J. T. (2011). An assessment of behavioral dynamic information processing measures in audiovisual speech perception. Front. Psychol. 2:238 10.3389/fpsyg.2011.00238 21980314 PMC3180170

[B6] Arnal L. H. Giraud A. L. (2012). Cortical oscillations and sensory predictions. Trends Cogn. Sci. 16, 390–398 10.1016/j.tics.2012.05.003 22682813

[B7] Arnal L. Morillon B. Kell C. Giraud A. (2009). Dual neural routing of visual facilitation in speech processing. J. Neurosci. 29, 13445–13453 10.1523/JNEUROSCI.3194-09.2009 19864557 PMC6665008

[B9] Arnal L. H. Wyart V. Giraud A. L. (2011). Transitions in neural oscillations reflect prediction errors generated in audiovisual speech. Nat. Neurosci. 14, 797–801 10.1038/nn.2810 21552273

[B10] Auer E. J. (2002). The influence of the lexicon on speech read word recognition: contrasting segmental and lexical distinctiveness. Psychon. Bull. Rev. 9, 341–347 10.3758/BF03196291 12120798

[B11] Bahrick L. E. (1992). Infant's perceptual differentiation of amodal and modaliy-specific audio-visual realations. J. Exp. Child Psychol. 53, 180–199 10.1016/0022-0965(92)90048-B 1578197

[B12] Balkany T. J. Hodges A. V. Eshraghi A. A. Butts S. Bricker K. Lingvai J. (2002). Cochlear implants in children-a review. Acta Otolaryngol. 122, 356–362 10.1080/00016480260000012 12125989

[B13] Barlow H. (1961). “Possible principles underlying the transformations of sensory messages,” in Sensory Communication, ed Rosenblith W. (Cambridge: MIT Press), 217–234

[B14] Barlow H. (1990). Conditions for versatile learning, Helmholtz's unconscious inference, and the task of perception. Vision Res. 30, 1561–1571 10.1016/0042-6989(90)90144-A 2288075

[B15] Barlow H. Földiak P. (1989). “Adaptation and decorrelation in the cortex,” in The Computing Neuron, eds Durbin R. Miall C. Mitchison G. (Wokingham: Addison-Wesley), 54–72

[B16] Barraclough N. E. Xiao D. Baker C. I. Oram M. W. Perrett D. I. (2005). Integration of visual and auditory information by superior temporal sulcus neurons responsive to the sight of actions. J. Cogn. Neurosci. 17, 377–391 10.1162/0898929053279586 15813999

[B17] Beauchamp M. S. Argall B. D. Bodurka J. Duyn J. H. Martin A. (2004). Unraveling multisensory integration: patchy organization within human STS multisensory cortex. Nat. Neurosci. 7, 1190–1192 10.1038/nn1333 15475952

[B18] Beauchamp M. S. Nath A. R. Pasalar S. (2010). fMRI-Guided transcranial magnetic stimulation reveals that the superior temporal sulcus is a cortical locus of the McGurk effect. J. Neurosci. 30, 2414–2417 10.1523/JNEUROSCI.4865-09.2010 20164324 PMC2844713

[B19] Bergeson T. R. Pisoni D. B. (2004). “Audiovisual speech perception in deaf adults and children following cochlear implantation,” in Handbook of Multisensory Integration, eds Calvert G. Spence C. Stein B. E. (Cambridge, MA: MIT Press), 749–772

[B20] Bergeson T. R. Pisoni D. B. Davis R. A. (2003). A longitudinal study of audiovisual speech perception by children with hearing loss who have cochlear implants. Volta Rev. 103, 347–370 21743753 PMC3130603

[B21] Bergeson T. R. Pisoni D. B. Davis R. A. (2005). Development of audiovisual comprehension skills in prelingually deaf children with cochlear implants. Ear Hear. 26, 149–164 10.1097/00003446-200504000-00004 15809542 PMC3432935

[B22] Bernstein L. Auer E. J. Wagner M. Ponton C. (2008). Spatiotemporal dynamics of audiovisual speech processing. Neuroimage 39, 423–435 10.1016/j.neuroimage.2007.08.035 17920933 PMC2185744

[B23] Besle J. Fischer C. Bidet-Caulet A. Lecaignard F. Bertrand O. Giard M. H. (2008). Visual activation and audiovisual interactions in the auditory cortex during speech perception: intracranial recordings in humans. J. Neurosci. 28, 14301–14310 10.1523/JNEUROSCI.2875-08.2008 19109511 PMC6671467

[B24] Besle J. Fort A. Delpuech C. Giard M.-H. (2004). Bimodal speech: early suppressive visual effects in human auditory cortex. Eur. J. Neurosci. 20, 2225–2234 10.1111/j.1460-9568.2004.03670.x 15450102 PMC1885424

[B25] Brancazio L. (2004). Lexical influences in audiovisual speech perception. J. Exp. Psychol. Hum. Percept. Perform. 30, 445–463 10.1037/0096-1523.30.3.445 15161378

[B26] Brungart D. Iyer N. Simpson B. van Wassenhove V. (2008). “The effects of temporal asynchrony on the intelligibility of accelerated speech,” in International Conference on Auditory-Visual Speech Processing (AVSP), (Moreton Island, QLD: Tangalooma Wild Dolphin Resort).

[B27] Brungart D. van Wassenhove V. Brandewie E. Romigh G. (2007). “The effects of temporal acceleration and deceleration on auditory-visual speech perception,” in International Conference on Auditory-Visual Speech Processing (AVSP) (Hilvarenbeek).

[B28] Busch N. A. VanRullen R. (2010). Spontaneous EEG oscillations reveal periodic sampling of visual attention. Proc. Natl. Acad. Sci. U.S.A. 107, 16048–16053 10.1073/pnas.1004801107 20805482 PMC2941320

[B29] Bushara K. O. Grafman J. Hallett M. (2001). Neural correlates of auditory-visual stimulus onset asynchrony detection. J. Neurosci. 21, 300–304 11150347 10.1523/JNEUROSCI.21-01-00300.2001 PMC6762435

[B30] Callan D. E. Jones J. A. Munhall K. G. Kroos C. Callan A. M. Vaitikiosis-Bateson E. (2004). Multisensory integrtaion sites identified by perception of spatial wavelet filtered visual speech gesture information. J. Cogn. Neurosci. 16, 805–816 10.1162/089892904970771 15200708

[B31] Callan D. Jones J. Munhall K. Callan A. Kroos C. Vatikiotis-Bateson E. (2003). Neural processes underlying perceptual enhancement by visual speech gestures. Neuroreport 14, 2213–2218 10.1097/00001756-200312020-00016 14625450

[B32] Calvert G. A. (1997). Activation of auditory cortex during silent lipreading. Science 276, 893–596 10.1126/science.276.5312.593 9110978

[B33] Calvert G. A. Brammer M. J. Bullmore E. T. Campbell R. Iversen S. D. David A. (1999). Response amplification in sensory-specific cortices during cross-modal binding. Neuroreport 10, 2619–2623 10.1097/00001756-199908200-00033 10574380

[B34] Calvert G. A. Campbell R. (2003). Reading speech from still and moving faces: the neural substrates of visible speech. J. Cogn. Neurosci. 15, 57–70 10.1162/089892903321107828 12590843

[B35] Calvert G. A. Campbell R. Brammer M. J. (2000). Evidence from functional magnetic resonance imaging of crossmodal binding in the human heteromodal cortex. Curr. Biol. 10, 649–657 10.1016/S0960-9822(00)00513-3 10837246

[B36] Calvert G. A. Thesen T. (2004). Multisensory integratio: methodological approaches and emerging principles in the human brain. J. Physiol. Paris 98, 191–205 10.1016/j.jphysparis.2004.03.018 15477032

[B37] Campbell R. (1986). Face recognition and lipreading. Brain 109, 509–521 10.1093/brain/109.3.509 3719288

[B38] Campbell R. (1989). “Lipreading,” in Handbook of Research on Face Processing, eds Young A. W. Ellis H. D. (Malden: Blackwell Publishing), 187–233

[B39] Campbell R. (1992). “Lip-reading and the modularity of cognitive function: neuropsychological glimpses of fractionation from speech and faces,” in Analytic Approaches to Human Cognition, eds Alegria J. Holender D. Junca de Morais J. Radeau M. (Amsterdam: Elsevier Science Publishers), 275–289

[B40] Campbell R. (2008). The processing of audio-visual speech: empirical and neural bases. Philos. Trans. R. Soc. Lond. B Biol. Sci. 363, 1001–1010 10.1098/rstb.2007.2155 17827105 PMC2606792

[B41] Campbell R. Garwood J. Franklinavi S. Howard D. Landis T. Regard M. (1990). Neuropsychological studies of auditory-visual fusion illusions. Four case studies and their implications. Neuropsychologia 28, 787–802 10.1016/0028-3932(90)90003-7 1701035

[B42] Campbell C. Massaro D. W. (1997). Perception of visible speech: influence of spatial quantization. Perception 26, 627–644 10.1068/p260627 9488886

[B43] Cathiard M.-A. Abry C. (2007). “Speech structure decisions from speech motion coordinations,” in Proceedings of the XVIth International Congress of Phonetic Sciences, Saarbrücken

[B44] Chandrasekaran C. Trubanova A. Stillittano S. Caplier A. Ghazanfar A. A. (2009). The natural statistics of audiovisual speech. PLoS Comput. Biol. 5:e1000436 10.1371/journal.pcbi.1000436 19609344 PMC2700967

[B45] Chomsky N. (2000). “Recent contributions to the theory of innate ideas,” in Minds, Brains and Computers The foundation of Cognitive Science, an Anthology, eds Harnish R. M. Cummins D. D. (Malden, MA: Blackwell), 452–457

[B46] Chomsky N. Halle M. (1968). The Sound Pattern of English. New York; Evanston; London: Harper and Row

[B47] Colin C. Radeau M. Soquet A. Demolin D. Colin F. Deltenre P. (2002). Mismatch negativity evoked by the McGurk-MacDonald effect: a phonetic representation within short-term memory. Clin. Neurophysiol. 113, 495–506 10.1016/S1388-2457(02)00024-X 11955994

[B48] Colonius H. Diederich A. (2004). Multisensory interaction in saccadic reaction time: a time-window-of-integration model. J. Cogn. Neurosci. 16, 1000–1009 10.1162/0898929041502733 15298787

[B49] Conrey B. Pisoni D. B. (2006). Auditory-visual speech perception and synchrony detection for speech and non speech signals. J. Acoust. Soc. Am. 119, 4065 10.1121/1.2195091 16838548 PMC3314884

[B50] Czigler I. Winkler I. Pató L. Várnagy A. Weisz J. Balázs L. (2006). Visual temporal window of integration as revealed by the visual mismatch negativity event-related potential to stimulus omissions. Brain Res. 1104, 129–140 10.1016/j.brainres.2006.05.034 16822480

[B51] de Gelder B. Böcker K. B. E. Tuomainen J. Hensen M. Vroomen J. (1999). The combined perception of emotion from voice and face: early interaction revealed by human electric brain responses. Neurosci. Lett. 260, 133–136 10.1016/S0304-3940(98)00963-X 10025717

[B52] Dehaene-Lambertz G. Dehaene S. Hertz-Pannier L. (2002). Functional neuroimaging of speech perception in infants. Science 298, 2013–2015 10.1126/science.1077066 12471265

[B53] Denève S. Pouget A. (2004). Bayesian multisensory integration and cross-modal spatial links. J. Neurophysiol. Paris 98, 249–258 10.1016/j.jphysparis.2004.03.011 15477036

[B54] Desimone R. Gross C. G. (1979). Visual areas in the temporal cortex of the macaque. Brain Res. 178, 363–380 10.1016/0006-8993(79)90699-1 116712

[B55] Driver J. Noesselt T. (2008). Multisensory interplay reveals crossmodal influences on ‘sensory-specific’ brain regions, neural responses, and judgments. Neuron 57, 11–23 10.1016/j.neuron.2007.12.013 18184561 PMC2427054

[B56] Erber M. P. (1978). Auditory-visual speech perception of speech with reduced optical clarity. J. Speech Hear. Res. 22, 213–223 491551 10.1044/jshr.2202.212

[B57] Ernst M. O. Bülthoff H. H. (2004). Meging the senses into a robust percept. Trends Cogn. Sci. 8, 162–169 10.1016/j.tics.2004.02.002 15050512

[B58] Evans K. K. Treisman A. (2010). Natural cross-modal mappings between visual and auditory features. J. Vis. 10:6 10.1167/10.1.6 20143899 PMC2920420

[B59] Friston K. (2005). A theory of cortical responses. Philos. Trans. R. Soc. Lond. B Biol. Sci. 360, 815–836 10.1098/rstb.2005.1622 15937014 PMC1569488

[B60] Ghazanfar A. A. Chandrasekaran C. Logothetis N. K. (2008). Interactions between the superior temporal sulcus and auditory cortex mediate dynamic face/voice integration in rhesus monkeys. J. Neurosci. 28, 4457–4469 10.1523/JNEUROSCI.0541-08.2008 18434524 PMC2663804

[B61] Ghazanfar A. A. Logothetis N. K. (2003). Facial expressions linked to monkey calls. Nature 423, 937–938 10.1038/423937a 12827188

[B62] Ghazanfar A. A. Schroeder C. E. (2006). Is neocortex essentially multisensory. Trends Cogn. Sci. 10, 278–285 10.1016/j.tics.2006.04.008 16713325

[B63] Ghazanfar A. A. Maier J. X. Hoffman K. L. Logothetis N. K. (2005). Multisensory integration of dynamic faces and voices in rhesus monkey auditory cortex. J. Neurosci. 25:5004 10.1523/JNEUROSCI.0799-05.2005 15901781 PMC6724848

[B64] Gibson E. J. (1969). Principles of Perceptual Learning and Development. New York, NY: Appleton - Century - Crofts

[B65] Giraud A. L. Poeppel D. (2012). Cortical oscillations and speech processing: emerging computational principles and operations. Nat. Neurosci. 15, 511–517 10.1038/nn.3063 22426255 PMC4461038

[B66] Grant K. W. (2002). Measures of auditory-visual integration for speech understanding: a theoretical perspective. J. Acoust. Soc. Am. 112, 30–33 10.1121/1.1482076 12141356

[B67] Grant K. W. Greenberg S. (2001). “Speech intelligibility derived from asynchronous processing of auditory-visual information,” in Auditory-Visual Speech Pocessing, (Aalborg).

[B68] Grant K. W. Seitz P. F. (1998). Measures of auditory-visual integration in nonsense syllables and sentences. J. Acoust. Soc. Am. 104, 2438–2450 10.1121/1.423751 10491705

[B69] Grant K. W. Seitz P.-F. (2000). The use of visible speech cues for improving auditory detection of spoken sentences. J. Acoust. Soc. Am. 108, 1197–1207 10.1121/1.1288668 11008820

[B70] Grant K. W. Walden B. E. Seitz P.-F. (1998). Auditory-visual speech recognition by hearing-impaired subjects: consonant recognition, sentence recognition and auditory-visual integration. J. Acoust. Soc. Am. 103, 2677–2690 10.1121/1.422788 9604361

[B71] Green K. P. (1996). “The use of auditory and visual information in phonetic perception,” in Speechreading by Humans and Machines, eds Stork D. G. Hennecke M. E. (Berlin: Springer-Verlag), 55–77

[B72] Greenberg S. (1998). A syllabic-centric framework for the evolution of spoken language. Brain Behav. Sci. 21, 267–268 10.1017/S0140525X98311176

[B73] Grossman E. Donnelly M. Price R. Pickens D. Morgan V. Neighbor G. (2000). Brain areas involved in perception of biological motion. J. Cogn. Neurosci. 12, 711–720 10.1162/089892900562417 11054914

[B74] Halle M. Stevens K. N. (1962). Speech recognition: a model and a program for research. IRE Trans. Inf. Theor. 8, 155–159 10.1109/TIT.1962.1057686

[B75] Hans-Otto K. (2001). New insights into the functions of the superior temporal cortex. Nat. Neurosci. 2, 568 10.1038/35086057 11484000

[B76] Harth E. Unnnikrishnan K. P. Pandya A. S. (1987). The inversion of sensory processing by feedback pathways: a model of visual cognitive functions. Science 237, 184–187 10.1126/science.3603015 3603015

[B77] Hasson U. Skipper J. Nusbaum H. Small S. (2007). Abstract coding of audiovisual speech: beyond sensory representation. Neuron 56, 1116–1126 10.1016/j.neuron.2007.09.037 18093531 PMC2175551

[B78] Hosoya T. Baccus S. A. Meister M. (2005). Dynamic predictive coding by the retina. Nature 436, 71–77 10.1038/nature03689 16001064

[B79] Houde J. F. Jordan M. I. (1998). Sensorimotor adaptation in speech production. Science 279, 1213–1216 10.1126/science.279.5354.1213 9469813

[B80] Jääskeläinen I. P. Ojanen V. Ahveninen J. Auranen T. Levänen S. Möttönen R. (2004). Adaptation of neuromagnetic N1 responses to phonetic stimuli by visual speech in humans. Neuroreport 18, 2741–2744 15597045

[B81] Jones J. Callan D. (2003). Brain activity during audiovisual speech perception: an fMRI study of the McGurk effect. Neuroreport 14, 1129–1133 10.1097/00001756-200306110-00006 12821795

[B82] Jordan T. R. McCotter M. V. Thomas S. M. (2000). Visual and audiovisual speech perception with color and gray-scale facial images. Percept. Psychophys. 62, 1394–1404 10.3758/BF03212141 11143451

[B83] Kayser C. Logothetis N. K. (2009). Directed interactions between auditory and superior temporal cortices and their role in sensory integration. Front. Integr. Neurosci. 3:7 10.3389/neuro.07.007.2009 19503750 PMC2691153

[B84] Kayser C. Logothetis N. K. Panzeri S. (2010). Visual enhancement of the information representation in auditory cortex. Curr. Biol. 20, 19–24 10.1016/j.cub.2009.10.068 20036538

[B85] Kayser C. Petkov C. I. Augath M. Logothetis N. K. (2007). Functional imaging reveals visual modulation of specific fields in auditory cortex. J. Neurosci. 27, 1824 10.1523/JNEUROSCI.4737-06.2007 17314280 PMC6673538

[B86] Kent R. D. (1983). “The segmental organization of speech, Chapter 4,” in The Production of Speech, ed. MacNeilage P. F. (Newyork, NY: Springer-verlag), 57–89

[B87] Kiebel S. J. Daunizeau J. Friston K. J. (2008). A hierarchy of time-scales and the brain. PLoS Comput. Biol. 4:e1000209 10.1371/journal.pcbi.1000209 19008936 PMC2568860

[B88] Kihlstrom J. F. (1987). The cognitive unconscious. Science 237, 1445–1452 10.1126/science.3629249 3629249

[B89] Kösem A. van Wassenhove V. (2012). Temporal structure in audiovisual sensory selection. PLoS ONE 7:e40936 10.1371/journal.pone.0040936 22829899 PMC3400621

[B90] Kuhl P. Meltzoff A. (1982). The bimodal perception of speech in infancy. Science 218, 1138–1141 10.1126/science.7146899 7146899

[B91] Kuhl P. Meltzoff A. N. (1984). The intermodal representation of speech in infants. Infant Behav. Dev. 7, 361–381 10.1016/S0163-6383(84)80050-8

[B92] Lakatos P. Karmos G. Mehta A. Ulbert I. Schroeder C. (2008). Entrainment of neuronal oscillations as a mechanism of attentional selection. Science 320, 110–113 10.1126/science.1154735 18388295

[B94] Lau E. F. Phillips C. Poeppel D. (2008). A cortical network for semantics: (de)constructing the N400. Nat. Rev. Neurosci. 9, 920–933 10.1038/nrn2532 19020511

[B95] Lewicki M. S. (2002). Efficient coding of natural sounds. Nat. Neurosci. 5, 356–363 10.1038/nn831 11896400

[B96] Lewkowicz D. J. (2000). The development of intersensory temporal perception: an epignetic systems/limitations view. Psychol. Bull. 162, 281–308 10.1037/0033-2909.126.2.281 10748644

[B97] Liberman A. M. Cooper F. S. Shankweiler D. P. Studdert-Kennedy M. (1967). Perception of the speech code. Psychol. Rev. 74, 431–461 10.1037/h0020279 4170865

[B98] Liberman A. M. Mattingly I. G. (1985). The motor theory of speech perception revised. Cognition 21, 1–36 10.1016/0010-0277(85)90021-6 4075760

[B99] Liégeois C. de Graaf J. B. Laguitton V. Chauvel P. (1999). Specialization of left auditory cortex for speech perception in man depends on temporal coding. Cereb. Cortex 9, 484–496 10.1093/cercor/9.5.484 10450893

[B100] Luo H. Liu Z. Poeppel D. (2010). Auditory cortex tracks both auditory and visual stimulus dynamics using low-frequency neuronal phase modulation. PLoS Biol. 8:e1000445 10.1371/journal.pbio.1000445 20711473 PMC2919416

[B101] Ma W. J. Beck J. M. Latham P. E. Pouget A. (2006). Bayesian inference with probabilistic population codes. Nat. Neurosci. 9, 1432–1438 10.1038/nn1790 17057707

[B102] MacDonald J. McGurk H. (1978). Visual influences on speech perception processes. Percept. Psychophys. 24, 253–257 10.3758/BF03206096 704285

[B103] MacDonald J. Soren A. Bachmann T. (2000). Hearing by eye: how much spatial degradation can be tolerated. Perception 29, 1155–1168 10.1068/p3020 11220208

[B104] MacKay D. M. (1958). Perceptual stability of a stroboscopically lit visual field containing self-luminous objects. Nature 181, 507–508 10.1038/181507a0 13517199

[B105] MacLeod A. Summerfield Q. (1987). Quantifying the contribution of vision to speech perception in noise. Br. J. Audiol. 21, 131–141 10.3109/03005368709077786 3594015

[B106] Maeda F. Kanai R. Shimojo S. (2004). Changing pitch induced visual motion illusion. Curr. Biol. 14, R990–R991 10.1016/j.cub.2004.11.018 15589145

[B107] Maier J. X. Di Luca M. Noppeney U. (2011). Audiovisual asynchrony detection in human speech. J. Exp. Psychol. Hum. Percept. Perform. 37, 245–256 10.1037/a0019952 20731507

[B108] Maiste A. C. Wiens A. S. Hunt M. J. Sherg M. Picton T. W. (1995). Event-related potentials and the categorical perception of speech sounds. Ear Hear. 16, 68–90 10.1097/00003446-199502000-00006 7774771

[B109] Martin B. Giersch A. Huron C. van Wassenhove V. (2012). Temporal event structure and timing in schizophrenia: preserved binding in a longer “now”. Neuropsychologia 51, 358–371 10.1016/j.neuropsychologia.2012.07.002 22813430

[B110] Massaro D. W. (1987). Speech Perception by Ear and Eye: a Paradigm for Psychological Inquiry. Hillsdale, NJ: Lawrence Erlbaum Associates, Inc

[B111] Massaro D. W. (1998). Perceiving Talking Faces. Cambridge: MIT Press

[B112] Massaro D. W. Cohen M. M. Smeele P. M. T. (1996). Perception of asynchronous and conflicting visual and auditory speech. J. Acoust. Soc. Am. 100, 1777 10.1121/1.417342 8817903

[B113] McGurk H. MacDonald J. (1976). Hearing lips and seeing voices. Nature 264, 746–748 10.1038/264746a0 1012311

[B114] Meltzoff A. N. (1999). Origins of theory of mind, cognition and communication. J. Commun. Disord. 32, 251–226 10.1016/S0021-9924(99)00009-X 10466097 PMC3629913

[B115] Meltzoff A. N. Moore M. K. (1979). Interpreting “imitative” responses in early infancy. Science 205, 217–219 10.1126/science.451596 451596

[B116] Miller L. D'Esposito M. (2005). Perceptual fusion and stimulus coincidence in the cross-modal integration of speech. J. Neurosci. 25, 5884–5893 10.1523/JNEUROSCI.0896-05.2005 15976077 PMC6724802

[B117] Morillon B. Lehongre K. Frackowiak R. S. J. Ducorps A. Kleinschmidt A. Poeppel D. (2010). Neurophysiological origin of human brain asymmetry for speech and language. Proc. Natl. Acad. Sci. U.S.A. 107, 18688–18693 10.1073/pnas.1007189107 20956297 PMC2972980

[B118] Möttönen R. Krause C. Tiippana K. Sams M. (2002). Processing of changes in visual speech in the human auditory cortex. Brain Res. Cogn. Brain Res. 13, 417–425 10.1016/S0926-6410(02)00053-8 11919005

[B119] Möttönen R. Schürmann M. Sams M. (2004). Time course of multisensory interactions during audiovisual speech perception in humans: a magnetoencephalographic study. Neurosci. Lett. 363, 112–115 10.1016/j.neulet.2004.03.076 15172096

[B120] Murray M. M. Spierer L. (2011). Multisensory integration: what you see is where you hear. Curr. Biol. 21, R229–R231 10.1016/j.cub.2011.01.064 21419991

[B121] Näätänen R. (1995). The mismatch negativity: a powerful tool for cognitive neuroscience. Ear Hear. 16, 6–18 10.1097/00003446-199502000-00002 7774770

[B122] Näätänen R. Gaillard A. W. Mäntysalo S. (1978). Early selective-attention effect on evoked potential reinterpreted. Acta Psychol. 42, 313–329 10.1016/0001-6918(78)90006-9 685709

[B123] Niparko J. K. Tobey E. A. Thal D. J. Eisenberg L. S. Wang N. Y. Quittner A. L. (2010). Spoken language development in children following cochlear implantation. JAMA 303, 1498–1506 10.1001/jama.2010.451 20407059 PMC3073449

[B124] Olson I. Gatenby J. Gore J. (2002). A comparison of bound and unbound audio-visual information processing in the human cerebral cortex. Brain Res. Cogn. Brain Res. 14, 129–138 10.1016/S0926-6410(02)00067-8 12063136

[B125] Panzeri S. Brunel N. Logothetis N. K. Kayser C. (2010). Sensory neural codes using multiplexed temporal scales. Trends Neurosci. 33, 111–120 10.1016/j.tins.2009.12.001 20045201

[B126] Paré M. Richler R. C. Ten Hove M. (2003). Gaze behavior in audiovisual speech perception: the influence of ocular fixations on the McGurk effect. Percept. Psychophys. 65, 553–567 10.3758/BF03194582 12812278

[B127] Pascual-Leone A. Hamilton R. (2001). The metamodal organization of the brain. Prog. Brain Res. 134, 427–445 10.1016/S0079-6123(01)34028-1 11702559

[B128] Philips C. Pellathy T. Marantz A. Yellin E. Wexler K. Poeppel D. (2000). Auditory cortex accesses phonological categories: an MEG mismatch study. J. Cogn. Neurosci. 12, 1038–1055 10.1162/08989290051137567 11177423

[B129] Piling M. (2009). Auditory event-related potentials (ERPs) in audiovisual speech perception. J. Speech Lang. Hear. Res. 52, 1073–1081 10.1044/1092-4388(2009/07-0276) 19641083

[B130] Poeppel D. (2003). The analysis of speech in different temporal integration windows: cerebral lateralization as asymmetric sampling in time. Speech Commun. 41, 245–255 10.1016/S0167-6393(02)00107-3

[B131] Poeppel D. Idsardi W. J. van Wassenhove V. (2008). Speech perception at the interface of neurobiology and linguistics. Philos. Trans. R. Soc. Lond. B Biol. Sci. 363, 1071–1086 10.1098/rstb.2007.2160 17890189 PMC2606797

[B132] Powers A. R. Hillock A. R. Wallace M. T. (2009). Perceptual training narrows the temporal window of multisensory binding. J. Neurosci. 29, 12265–12274 10.1523/JNEUROSCI.3501-09.2009 19793985 PMC2771316

[B133] Puce A. Allison T. Bentin A. Gore J. C. McCarthy G. (1998). Temporal cortex activation in humans viewing eye and mouth movements. J. Neurosci. 18, 2188–2199 9482803 10.1523/JNEUROSCI.18-06-02188.1998 PMC6792917

[B134] Pylyshyn Z. (1984). Computation and Cognition: Towards a Foundation for Cognitive Science. Cambridge: MIT Press

[B135] Rajkai C. Lakatos P. Chen C. Pincze Z. Karmos G. Schroeder C. (2008). Transient cortical excitation at the onset of visual fixation. Cereb. Cortex 18, 200–209 10.1093/cercor/bhm046 17494059

[B136] Rao R. P. N. Ballard D. H. (1999). Predictive coding in the visual cortex: a functional interpretation of some extra-classical receptive-field effects. Nat. Neurosci. 2, 79–87 10.1038/4580 10195184

[B137] Reale R. Calvert G. Thesen T. Jenison R. Kawasaki H. Oya H. (2007). Auditory-visual processing represented in the human superior temporal gyrus. Neuroscience 145, 162–184 10.1016/j.neuroscience.2006.11.036 17241747

[B138] Remez R. (2003). Establishing and maintaining perceptual coherence: unimodal and multimodal evidence. J. Phon. 31, 293–304 10.1016/S0095-4470(03)00042-1

[B139] Remez R. E. Fellowes J. M. Pisoni D. B. Goh W. D. Rubin P. E. (1998). Multimodal perceptual organization of speech: Evidence from tone analogs of spoken utterances. Speech Commun. 26, 65–73 10.1016/S0167-6393(98)00050-8 21423823 PMC3060793

[B140] Rosen S. (1992). temporal information in speech: acoustic, auditory and linguistic aspects. Philos. Trans. R. Soc. Lond. B 336, 367–373 10.1098/rstb.1992.0070 1354376

[B141] Rosenblum L. Schmuckler M. A. Johnson J. A. (1997). The McGurk effect in infants. Percept. Psychophys. 59, 347–357 10.3758/BF03211902 9136265

[B142] Rosenblum L. D. Saldaña H. M. (1996). An audiovisual test of kinematic primitives for visual speech perception. J. Exp. Psychol. Hum. Percep. Perform. 22, 318–331 10.1037/0096-1523.22.2.318 8934846

[B143] Rosenblum L. Yakel D. A. (2001). The McGurk effect from single and mixed speaker stimuli. Acoust. Res. Lett. Online 2, 67–72 10.1121/1.1366356

[B144] Saltzman E. L. Munhall K. G. (1989). A dynamical approach to gestural patterning in speech production. Ecol. Psychol. 1, 333–382 10.1207/s15326969eco0104_2

[B145] Sams M. Aulanko R. (1991). Seeing speech: visual information from lip movements modifies activity in the human auditory cortex. Neurosci. Lett. 127, 141–147 10.1016/0304-3940(91)90914-F 1881611

[B146] Schorr E. Fox N. van Wassenhove V. Knudsen E. (2005). Auditory-visual fusion in speech perception in children with cochlear implants. Proc. Natl. Acad. Sci. U.S.A. 102, 18748–18750 10.1073/pnas.0508862102 16339316 PMC1317952

[B147] Schroeder C. Lakatos P. (2009). Low-frequency neuronal oscillations as instruments of sensory selection. Trends Neurosci. 32, 9–18 10.1016/j.tins.2008.09.012 19012975 PMC2990947

[B148] Schroeder C. Lakatos P. Kajikawa Y. Partan S. Puce A. (2008). Neuronal oscillations and visual amplification of speech. Trends Cogn. Sci. 12, 106–113 10.1016/j.tics.2008.01.002 18280772 PMC3987824

[B149] Schwartz J. Robert-Ribes J. Escudier P. (1998). “Ten years after summerfield: a taxonomy of models for audio-visual fusion in speech perception,” in Hearing by Eye II: Advances in the Psycholoy of Speechreading and Auditory-Visual Speech, eds Campbell R. Dodd B. Burnham D. (East Sussex: Psychology Press), 85–108

[B150] Schwartz J.-L. (2003). “Why the FLMP should not be applied to McGurk data…or how to better compare models in the Bayesian framework,” in AVSP - International Conference on Audio-Visual Speech Processing, (St-Jorioz).

[B151] Schwartz J.-L. Basirat A. Ménard L. Sato M. (2012). The Perception-for-Action-Control Theory (PACT): A perceptuo-motor theory of speech perception. J. Neurolinguistics 25, 336–354 10.1016/j.jneuroling.2009.12.004

[B152] Scott S. K. McGettigan C. Eisner F. (2009). A little more conversation, a little less action-candidate roles for the motor cortex in speech perception. Nat. Rev. Neurosci. 10, 295–302 10.1038/nrn2603 19277052 PMC4238059

[B153] Sekiyama K. (1994). Differences in auditory-visual speech perception between Japanese and americans: McGurk effect as a function of incompatibility. J. Acoust. Soc. Am. 15, 143–158 10.1250/ast.15.143

[B154] Sekiyama K. (1997). Cultural and linguistic factors in audiovisual speech processing: the McGurk effect in Chinese subjects. Percept. Psychophys. 59, 73–80 10.3758/BF03206849 9038409

[B155] Sekiyama K. Kanno I. Miura S. Sugita Y. (2003). Auditory-visual speech perception examined by fMRI and PET. Neurosci. Res. 47, 277–287 10.1016/S0168-0102(03)00214-1 14568109

[B156] Sekiyama K. Tohkura Y. (1991). McGurk effect in non-English listeners: Few visual effects for Japanese subjects hearing Japanese syllables of high auditory intelligibility. J. Acoust. Soc. Am. 90, 1797–1805 10.1121/1.401660 1960275

[B157] Servos P. Osu R. Santi A. Kawato M. (2002). The neural substrates of biological motion perception: an fMRI study. Cereb. Cortex 12, 772–782 10.1093/cercor/12.7.772 12050089

[B158] Sharma A. Dorman M. F. (1999). Cortical auditory evoked potential correlates of categorical perception of voice-onset time. J. Acoust. Soc. Am. 16, 1078–1083 10.1121/1.428048 10462812

[B159] Sharma A. Dorman M. F. Spahr A. J. (2002). Rapid development of cortical auditory evoked potentials after early cochlear implantation. Neuroreport 13, 1365–1368 10.1097/00001756-200207190-00030 12151804

[B160] Sharma J. Dragoi V. Tenebaum J. B. (2003). V1 neurons signal acquisition of an internal representation of stimulus location. Science 300, 1758–1763 10.1126/science.1081721 12805552

[B161] Simos P. G. Diehl R. L. Breier J. I. Molis M. R. Zouridakis G. Papanicolaou A. C. (1998). MEG correlates of categorical perception of a voice onset time continuum in humans. Cogn. Brain Res. 7, 215–219 10.1016/S0926-6410(98)00037-8 9774735

[B162] Skipper J. van Wassenhove V. Nusbaum H. Small S. (2007). Hearing lips and seeing voices: How cortical areas supporting speech production mediate audiovisual speech perception. Cereb. Cortex 17, 2387–2399 10.1093/cercor/bhl147 17218482 PMC2896890

[B163] Smith E. C. Lewicki M. S. (2006). Efficient auditory coding. Nature 439, 978–982 10.1038/nature04485 16495999

[B164] Soto-Faraco S. Navarra J. Alsius A. (2004). Assessing automaticity in audiovisual speech integration: evidence from the speeded classification task. Cognition 92, B13–B23 10.1016/j.cognition.2003.10.005 15019556

[B165] Spelke E. S. (1981). The infant's acquisition of knowledge of bimodally specified events. J. Exp. Child Psychol. 31, 279–299 10.1016/0022-0965(81)90018-7 7217891

[B166] Srinivasan M. V. Laughlin S. B. Dubs A. (1982). Predictive coding: a fresh view of inhibition in the retina. Proc. R. Soc. Lond. B Biol. Sci. 216, 427–459 10.1098/rspb.1982.0085 6129637

[B167] Stekelenburg J. Vroomen J. (2007). Neural correlates of multisensory integration of ecologically valid audiovisual events. J. Cogn. Neurosci. 19, 1964–1973 10.1162/jocn.2007.19.12.1964 17892381

[B168] Stekelenburg J. J. Vroomen J. (2012). Electrophysiological evidence for a multisensory speech-specific mode of perception. Neuropsychologia 50, 1425–1431 10.1016/j.neuropsychologia.2012.02.027 22410413

[B169] Stevens K. (1960). Toward a model of speech perception. J. Acoust. Soc. Am. 32, 45–55 10.1121/1.1907874

[B170] Stevenson R. A. Altieri N. A. Kim S. Pisoni D. B. James T. W. (2010). Neural processing of asynchronous audiovisual speech perception. Neuroimage 49, 3308–3318 10.1016/j.neuroimage.2009.12.001 20004723 PMC2818746

[B171] Stevenson R. A. Van DerKlok R. M. Pisoni D. B. James T. W. (2011). Discrete neural substrates underlie complementary audiovisual speech integration processes. Neuroimage 55, 1339–1345 10.1016/j.neuroimage.2010.12.063 21195198 PMC3057325

[B172] Stevenson R. A. Zemtsov R. K. Wallace M. T. (2012). Individual differences in the multisensory temporal binding window predict susceptibility to audiovisual illusions. J. Exp. Psychol. Hum. Percept. Perform. 38, 1517–1529 10.1037/a0027339 22390292 PMC3795069

[B173] Sumby W. Pollack I. (1954). Visual contribution to speech intelligibility in noise. J. Acoust. Soc. Am. 26, 212–215 10.1121/1.1907309

[B174] Summerfield A. Q. (1987). “Some preliminaries to a comprehensive account of audio-visual speech perception,” in Hearing by Eye, eds Dodd B. Campbell R. (London: Erlbaum Associates), 3–51

[B175] Svirsky M. A. Robbins A. M. Kirk K. I. Pisoni D. B. Miyamoto R. T. (2000). Language development in profoundly deaf children with cochlear implants. Psychol. Sci. 11, 153–158 10.1111/1467-9280.00231 11273423 PMC3429133

[B176] Talsma D. Senkowski D. Soto-Faraco S. Woldorff M. G. (2010). The multifaceted interplay between attention and multisensory integration. Trends Cogn. Sci. 14, 400–410 10.1016/j.tics.2010.06.008 20675182 PMC3306770

[B177] Tervaniemi M. Maury S. Näätänen R. (1994). Neural representations of abstract stimulus features in the human brain as reflected by the mismatch negativity. Neuroreport 5, 844–846 10.1097/00001756-199403000-00027 8018861

[B178] Theunissen F. Miller J. P. (1995). Temporal encoding in nervous systems: a rigorous definition. J. Comput. Neurosci. 2, 149–162 10.1007/BF00961885 8521284

[B179] Tiippana K. Andersen T. S. Sams M. (2003). Visual attention modulates audiovisual speech perception. Eur. J. Cogn. Psychol. 16, 457–472 10.1080/0954144034000026822623217

[B180] Todorovic A. de Lange F. P. (2012). Repetition suppression and expectation suppression are dissociable in time in early auditory evoked fields. J. Neurosci. 32, 13389–13395 10.1523/JNEUROSCI.2227-12.2012 23015429 PMC6621367

[B181] Tuller B. Kelso J. A. (1984). The timing of articulatory gestures: evidence for relational invariants. J. Acoust. Soc. Am. 76, 1030–1036 10.1121/1.391421 6501697

[B182] Tuomainen J. Andersen T. S. Tiippana K. Sams M. (2005). Audio-visual speech perception is special. Cognition 96, B13–B22 10.1016/j.cognition.2004.10.004 15833302

[B183] Vaina L. M. Solomon J. Chowdhury S. Sinha P. Belliveau J. W. (2001). Functional neuroanatomy of biological motion perception in humans. Proc. Natl. Acad. Sci. 98, 11656–11661 10.1073/pnas.191374198 11553776 PMC58785

[B184] van Wassenhove V. (2009). Minding time in an amodal representational space. Philos. Trans. R. Soc. B Biol. Sci. 364, 1815–1830 10.1098/rstb.2009.0023 19487185 PMC2685822

[B185] van Wassenhove V. Ghazanfar A. Munhall K. Schroeder C. (2012). “Bridging the gap between human and non human studies of audiovisual integration,” in The New Handbook of Multisensory Processing, ed Stein B. E. (Cambridge: MIT Press), 153–167

[B186] van Wassenhove V. Grant K. Poeppel D. (2007). Temporal window of integration in auditory-visual speech perception. Neuropsychologia 45, 598–607 10.1016/j.neuropsychologia.2006.01.001 16530232

[B187] van Wassenhove V. Grant K. W. Poeppel D. (2005). Visual speech speeds up the neural processing of auditory speech. Proc. Natl. Acad. Sci. U.S.A. 102, 1181–1186 10.1073/pnas.0408949102 15647358 PMC545853

[B188] Vatikiotis-Bateson E. Eigsti I.-M. Yano S. Munhall K. G. (1998). Eye movement of perceivers during audiovisual speech perception. Percept. Psychophys. 60, 926–940 10.3758/BF03211929 9718953

[B189] Viviani P. Figliozzi F. Lacquaniti F. (2011). The perception of visible speech: estimation of speech rate and detection of time reversals. Exp. Brain Res. 215, 141–161 10.1007/s00221-011-2883-9 21986668

[B190] Voss P. Zatorre R. J. (2012). Organization and reorganization of sensory-deprived cortex. Curr. Biol. 22, R168–R173 10.1016/j.cub.2012.01.030 22401900

[B191] Vroomen J. Keetels M. (2010). Perception of intersensory synchrony: a tutorial review. Atten. Percept. Psychophys. 72, 871–884 10.3758/APP.72.4.871 20436185

[B192] Wacongne C. Labyt E. van Wassenhove V. Bekinschtein T. Naccache L. Dehaene S. (2011). Evidence for a hierarchy of predictions and prediction errors in human cortex. Proc. Natl. Acad. Sci. U.S.A. 108, 20754–20759 10.1073/pnas.1117807108 22147913 PMC3251061

[B193] Walker S. Bruce V. O'Malley C. (1995). Facial identity and facial speech processing: Familiar faces and voices in the McGurk effect. Attent. Percept. Psychophys. 57, 1124–1133 10.3758/BF03208369 8539088

[B194] Walker-Andrews A. S. (1986). Intermodal perception of expressive behaviors: relation of eye and voice. Dev. Psychol. 22, 373–377 10.1037/0012-1649.22.3.373

[B195] Waltzman S. B. Cohen N. L. Gomolin L. H. Green J. E. Shapiro W. H. Hoffman R. A. (1997). Open-set speech perception in congenitally deaf children using cochlear implants. Am. J. Otol. 18, 342–349 9149829

[B196] Wang X. J. (2010). Neurophysiological and computational principles of cortical rhythms in cognition. Physiol. Rev. 90, 1195–1268 10.1152/physrev.00035.2008 20664082 PMC2923921

[B197] Werner-Reiss U. Kelly K. Trause A. Underhill A. Groh J. (2003). Eye position affects activity in primary auditory cortex of primates. Curr. Biol. 13, 554–562 10.1016/S0960-9822(03)00168-4 12676085

[B198] Wright T. Pelphrey K. Allison T. McKeown M. McCarthy G. (2003). Polysensory interactions along lateral temporal regions evoked by audiovisual speech. Cereb. Cortex 13, 1034–1043 10.1093/cercor/13.10.1034 12967920

[B199] Wundt W. (1874). Grundzuge Derphysiologischen Psychologie, Leipzig: Engelmann

[B200] Yabe H. Tervaniemi M. Reinikainen K. Näätänen R. (1997). Temporal window of integration revealed by MMN to sound omission. Neuroreport 8, 1971–1974 10.1097/00001756-199705260-00035 9223087

[B201] Yuille A. Kersten D. (2006). Vision as Bayesian inference: analysis by synthesis. Trends Cogn. Sci. 10, 301–308 10.1016/j.tics.2006.05.002 16784882

[B202] Zion Golumbic E. Cogan G. B. Schroeder C. E. Poeppel D. (2013). Visual input enhances selective speech envelope tracking in auditory cortex at a “cocktail party”. J. Neurosci. 33, 1417–1426 10.1523/JNEUROSCI.3675-12.2013 23345218 PMC3711546

